# Natural *Besnoitia besnoiti* infections in cattle: chronology of disease progression

**DOI:** 10.1186/s12917-015-0344-6

**Published:** 2015-02-14

**Authors:** Nicole S Gollnick, Julia C Scharr, Gereon Schares, Martin C Langenmayer

**Affiliations:** Clinic for Ruminants with Ambulatory and Herd Health Services at the Centre for Clinical Veterinary Medicine, Veterinary Faculty, Ludwig-Maximilians-Universitaet Muenchen, Sonnenstrasse 16, 85764 Oberschleissheim, Germany; 89129 Rammingen, Germany; Friedrich-Loeffler-Institut, Federal Research Institute for Animal Health, Institute of Epidemiology, Suedufer 10, 17493 Greifswald-Insel Riems, Germany; Institute of Veterinary Pathology at the Centre for Clinical Veterinary Medicine, Veterinary Faculty, Ludwig-Maximilians-Universitaet Muenchen, Veterinaerstr. 13, 80539 Munich, Germany

**Keywords:** *Besnoitia besnoiti*, Bovine besnoitiosis, Cattle, Natural infection, Acute disease, Chronic disease, Laminitis

## Abstract

**Background:**

Bovine besnoitiosis is an emerging protozoan disease in cattle. Neither vaccines nor chemotherapeutic drugs are currently available for prevention and treatment of *Besnoitia besnoiti* infections. Therefore the implementation of appropriate disease management strategies is of utmost importance. The aim of this longitudinal study was to complement current knowledge on the chronology of disease progression. This was realized by correlating clinical findings in early stages of naturally acquired bovine besnoitiosis with results of real-time PCR of skin biopsies and of two western immunoblots and an immunofluorescent antibody test (IFAT).

Animals for this study were obtained by i) closely monitoring a cow-calf operation with a high prevalence of bovine besnoitiosis for cases of acute disease, and by ii) conducting a 12-week cohabitation experiment on pasture with five healthy heifers, a healthy bull and five *B. besnoiti* infected cows. A control group of six healthy heifers was kept at a minimal distance of 20 m. Further, the spectrum of potential insect vectors was determined.

**Results:**

Infected cattle were followed up to a maximum of 221 days after first detection of *B. besnoiti* antibodies. Two severely affected cows developed visible and palpable alterations of skin, a decrease in body condition despite good feed intake, and chronic bovine besnoitiosis-associated laminitis leading to non-healing sole ulcers. The cows also had high reciprocal IFAT titers and high loads of parasite DNA in skin samples. Two heifers developed a mild clinical course characterized by few parasitic cysts visible in the scleral *conjunctivae* and *vestibula vaginae*. Both heifers became infected during the time of high insect activity of the species *Musca domestica*, *Musca autumnalis*, *Haematobia irritans*, and *Stomoxys calcitrans*. When a third heifer became subclinically infected, low insect activity was recorded. None of the six control heifers contracted a *B. besnoiti* infection.

**Conclusions:**

In chronic besnoitiosis, the severe clinical course apparently corresponded with high reciprocal IFAT titers and high loads of parasite DNA in skin, whereas mild and subclinical cases displayed lower values. Bovine besnoitiosis-associated laminitis represents an important complication in severe chronic disease which severely impairs animal welfare.

**Electronic supplementary material:**

The online version of this article (doi:10.1186/s12917-015-0344-6) contains supplementary material, which is available to authorized users.

## Background

Bovine besnoitiosis is caused by the apicomplexan parasite *Besnoitia besnoiti* [1]. Severe acute disease is characterized by fever, subcutaneous edema, conjunctivitis, nasal discharge, salivation, lameness, and depression [2-6]. In the chronic stage of bovine besnoitiosis, the parasite forms cysts in connective tissues, especially the dermis and the non-intestinal mucosa [5-8]. Of special diagnostic value are the superficially located cysts in the scleral *conjunctivae*, mucous membranes lining the nasal cavity and the *vestibulum vaginae* [9-11]. These pin-head sized, white protuberances are pathognomonic for bovine besnoitiosis [5]. In severe cases of the disease, the massive parasitism of the dermis also leads to visible and palpable changes of the skin. It becomes uneven and thicker, and disturbance of local blood perfusion may lead to alopecia and skin necrosis [5,8]. To date, vaccines and chemotherapeutical drugs for prevention and treatment of the disease are not available [6,12].

Cattle are considered to be intermediate hosts while the definitive host is still unknown [13,14]. Therefore, the complete life cycle of *B. besnoiti* remains yet to be elucidated. However, it has been established by experiments that hematophagous insects are able to transmit the parasite between cattle [2]. Further, the close contact of infected and healthy animals has been suggested to play a pivotal role in disease transmission [2,12].

Clinical and pathophysiological aspects of chronic bovine besnoitiosis are well described in the literature, as a number of such cases of naturally or experimentally acquired disease in cattle has been reported over the past century [8,10,11,15-25]. But especially studying the early stages of naturally acquired bovine besnoitiosis has proved to be difficult. This may be either due to the limitation of access to individual animals in extensive management systems where acute cases may go undetected or simply due to subclinical course of infections [2,25,26].

As bovine besnoitiosis is spreading within Europe, the demand for more scientific investigations is increasing [12,27]. Thus far, longitudinal studies focusing on early stages of naturally acquired bovine besnoitiosis combining the results of clinical examinations and current state-of-the-art laboratory tests are lacking [5]. Therefore, the objective of the present study was to augment current knowledge concerning the chronology of disease progression.

Animals for this study were obtained by i) closely monitoring a German cattle herd with a high prevalence of bovine besnoitiosis for cases of acute disease (Herd-BbGer1) [28], and by ii) conducting a cohabitation experiment involving healthy and *B. besnoiti* infected cattle. Clinical examinations were correlated with the results on antibody development and the detectability of *B. besnoiti* DNA over time in one of the parasite’s target organs, the skin.

## Methods

### Ethical statement

Permission for this study was granted by the responsible authorities (Animal ethics committee; Regional government of Upper Bavaria). The experiment was registered under TV Az. 55.2-54-2531-83-09. After completion of the cohabitation period, all animals remained on the premises for fattening or breeding purposes until submitted to slaughter or necropsy.

### Animals and experimental design

The study consisted of a 12-week cohabitation period (August 18, 2009, until November 9, 2009) and a five-month follow-up period. Six healthy Simmental heifers (Study animals [SA] 2, 5, 7, 10, 11, and 12) were randomly assigned to a paddock (control) group. Five healthy Simmental heifers (SA 3, 4, 6, 8, and 9) and a healthy Simmental bull (SA 1) were kept on a 2,500 m^2^ pasture together with three clinically infected non-pregnant Limousin cows (SA 13, 15, and 16) with a well documented history of chronic bovine besnoitiosis. These cows showed typical parasitic cysts in the scleral *conjunctivae* and *vestibula vaginae*, alterations of skin, and had tested positive in IFAT, immunoblot, histology and PCR (Additional file 1). Further, two pregnant Limousin cows (SA 20 and 22) in the acute stage of disease were added to the pasture group on trial day [td] 3 and 51, respectively. Both animals came from subsets of Herd-BbGer1 with *B. besnoiti* seroprevalence of 92.5% and 82.8%, respectively (data not shown). The minimal distance between both groups was 20 m. The age range of Simmental and Limousin cattle was 11 to 20 months and three to eight years, respectively. All cattle tested negative for *Neospora caninum* antibodies by an immunofluorescent antibody test (IFAT) and by immunoblots as previously described [29,30]. During a two-week quarantine period, claws of all Simmental cattle were trimmed and thorough clinical examinations were conducted daily.

Throughout the 12-week cohabitation period, social and reproductive behavior of study animals was recorded daily. This included heat detection in non-pregnant animals on pasture during the morning hours before, during, and after clinical examinations, and in the evening after feeding time. Mating activity of the bull was recorded. The non-pregnant females on pasture were treated regularly with 150 μg Cloprostenol i.m., a synthetic PGF2α analog (Dalmazin®, FATRO S.p.A., Ozzano Emilia, Italy) to induce estrus. For ovarian cyst treatment, 100 μg Lecirelin i.m., a synthetic GnRH analog was used (Dalmarelin®, FATRO S.p.A., Ozzano Emilia, Italy). In accordance with animal welfare regulations (see Ethical statement), it was required to treat animals showing signs of acute bovine besnoitiosis with 0.5 mg/kg of Meloxicam s.c. (Metacam®, Boehringer Ingelheim, Vetmedica GmbH, Ingelheim/Rhein, Germany). This treatment was repeated every 48 hours until clinical signs of disease subsided. Cattle with a lameness score of ≥ 3 underwent functional claw trimming and appropriate treatment. Deep claw ulcers were treated under intravenous regional analgesia using 20 ml of Procain-Hydrochlorid 2% (Procasel 2%®, Selectavet, Weyarn/Holzolling, Germany).

### Clinical examinations and samplings

Clinical examinations and samplings were performed in the morning while cattle were restrained in a cattle chute (paddock group) or in headlocks at the feed bunk (pasture group). See Table 1 for further details about the examination protocols ‘daily exam’ and ‘status exam’. Scores 1 (mild), 2 (moderate), and 3 (severe) were assigned to classify the degree of alterations. The body condition score (BCS) was determined according to Edmonson et al. [31]. For grading gait disturbances, the five-point scoring system described by Sprecher et al. (1997) was used [32]: Normal gait (1), mild (2), moderate (3), moderate to severe (4), and severe lameness (5). The number of cysts in the mucous membrane of the *vestibulum vaginae* and the mean number of cysts in the scleral *conjunctivae* were estimated and the result were assigned to the following categories: 1: 1–5 cysts; 2: 6–10 cysts; 3: 11–20 cysts; 4: 21–30 cysts; 5: >30 cysts.

**Table 1:** **Clinical examination protocols**

**Examination of**	**Daily exam protocol** ^**a**^	**Status exam protocol** ^**b,c**^
Temperament	●	●
Posture	●	●
Gait	●	●
Temperature	●	●
Body condition		●
Cardiovascular system	●	●
Respiratory tract	●	●
Digestive tract	●	●
Musculosceletal system	●	●
Female reproductive system		●
Skin		●
Palpable lymph nodes^d^		●
Abdominal cavity by rectal exploration		●
Scleral *conjunctivae*		●
Mucous membrane of *vestibulum vaginae*		●

^a^Used daily (except Mondays and Thursdays) for *Besnoitia besnoiti* negative Simmental cattle.

^b^Used Mondays and Thursdays for *B. besnoiti* negative Simmental cattle.

^c^Used daily for Simmentals after developing signs of clinical besnoitiosis until the end of the cohabitation experiment. Used daily for study animal 20, and for study animal 22 on trial days 51, 52, 56, 63, 70, and 80. Used on all animals examined during the five-month follow-up period.

^d^ *Lymphonodi [lnn.] mandibulares, lnn. parotidei, lnn. retropharyngei mediales, lnn. cervicales superficiales, lnn. subiliaci, lnn. ileofemorales*.

#### Blood and skin biopsy sampling

On td 0, blood samples were collected from all Simmentals and SA 13, 15, and 16. Further, skin biopsies for PCR (except SA 13, 15, and 16) and histological examinations were taken under local anesthesia with 8-mm punch biopsy devices at the femoral regions of the hind legs [33,34]. Thereafter, skin biopsies for PCR were taken on Mondays and Thursdays over the 12-week cohabitation period from healthy Simmental cattle. In case a *B. besnoiti* infection had been observed in an individual animal, blood samples were taken every day and skin samples were obtained at least every other day at the femoral region or, less often, laterally at the neck during 21 days after initial signs of acute disease. Thereafter, blood and skin samples were again taken twice a week. With regards to SA 20, the sampling regime for acutely infected cattle was applied. Due to its uncooperative behavior, SA 22 was only sampled on days 51, 52, 56, 63, 70, and 80. During regular herd visits in the five-month follow-up period, complete samplings were performed on SA 4, 6, 8, 20, and 22 every two to three weeks.

#### Serology and real-time PCR examinations

An immunofluorescent antibody test (IFAT) and two western immunoblots (one with tachyzoite and the second with bradyzoite antigen) were performed to define the serological status of the animals. The combination of results of these three serological tests is referred to as “serological reference system” later on. Thus, a blood sample was regarded as seropositive if it revealed a positive result in at least two of the three tests applied. In IFAT, a serum was regarded as positive if the reciprocal IFAT titer was ≥ 200. In the immunoblot, a positive serological reaction was recorded if ≥ 4 of 10 bands selected per antigen (tachyzoite, bradyzoite) were recognized [29]. Previously published real-time PCR results for the detection of a *B. besnoiti* infection were included for data interpretation [34]. For calculation of mean cycle threshold [ct]-values and statistical analysis, negative real-time PCR results were counted with a ct-value of 45.0.

### Examinations by light microscopy

Immediately after collection of blood into tubes precoated with EDTA, a blood smear was performed on a glass slide. Smears were stained with a Pappenheim stain (Haema Schnellfaerbung, LT-SYS® Labor + Technik Eberhard Lehmann GmbH, Berlin, Germany) and examined for *B. besnoiti* tachyzoites. At necropsy of SA 20 and 22, samples of the claws were fixed in paraformaldehyde 4% for 24 to 48 hours, decalcified for one week and subsequently embedded in plastic [35]. Sections of 2 μm thickness were routinely stained with hematoxylin and eosin (HE) and according to Giemsa.

### Insects: collection, DNA extraction and real-time PCR

Insects alighting on or flying in the vicinity of the trunk, neck and head of the pasture group cattle restrained in headlocks were caught using a Japan insect hand net on td 24 at 1 and 2 pm, and on td 32 at 11 am. Insects were stored in 96.5% ethanol. Using a dissecting microscope, insect classification was conducted based on morphological characteristics at the Institute for Parasitology and Tropical Veterinary Medicine, Berlin, Germany. Using a vacuum evaporator (Alpha-RVC, Martin Christ Gefriertrocknungsanlagen GmbH, Osterode am Harz, Germany), insects were dried for 60 min at 37°C. DNA extraction was performed using the ZR Insect/Tissue DNA Kit-25™ (ZYMO Research Corporation, Irvine, CA, USA) following the manufacturer’s procedure with a variation in bead beater process time (15 min instead of 10 min). Every 11th sample, a negative processing control (empty tube), was included during DNA extraction. All DNAs were tested by real-time PCR using the Bb-RT2 assay as previously described [36].

### Definitions

Acute, subacute, and chronic bovine besnoitiosis are characterized by a number of specific and non-specific clinical signs reviewed by [5,6]. In our study we chose specific clinical features for assigning a study animal to a respective disease stage: Animals in the acute stage of bovine besnoitiosis had to show fever (body temperature > 39.0°C) or at least two of the following clinical signs/diagnoses: depression, conjunctivitis, subcutaneous edema, lymphadenitis, lameness. Cattle were classified as chronically infected by *B. besnoiti* when first parasitic cysts were visible in the scleral *conjunctivae* or mucous membranes. The time period between the acute and chronic stage is referred to as subacute stage of disease. The assumed time period of infection is defined as the period of 11–14 days prior to the onset of clinical signs and was based on previous studies [2].

## Results

### New infections by *B. besnoiti*

**Figure 1:**
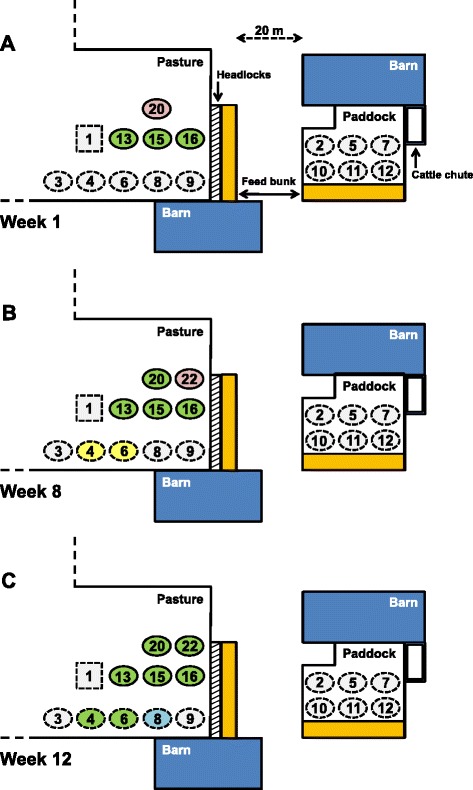
**Experimental design and results of cohabitation trial.** Sixteen female adult cattle (O) and one breeding bull (□) of the breeds Limousin (─) and German Simmental (−−−) were enrolled in the 12-week cohabitation trial. Pink, yellow, green, and blue indicate animals in the acute, subacute, chronic, and subclinical stage of bovine besnoitiosis, respectively. **A)** Five healthy, non-pregnant Simmental heifers (Study animals [SA] 3, 4, 6, 8, and 9), and a healthy Simmental bull (SA 1) were kept together with three chronically *Besnoitia besnoiti* infected, non-pregnant Limousin cows (SA 13, 15, and 16) on a 2,500 m^2^ pasture. A control group of six healthy, non-pregnant Simmental heifers (SA 2, 5, 7, 10, 11, and 12) were confined at a minimal distance of 20 m in a 200 m^2^ paddock area. On trial day [td] 3, the acutely *B. besnoiti* infected, pregnant Limousin cow SA 20 was introduced into the pasture group. **B)** The acutely *B. besnoiti* infected, pregnant Limousin cow SA 22 was introduced on td 51. By this time SA 20 had entered the chronic stage, and SA 4 and 6 were in the subacute stage of disease. **C)** In the final week of the trial, SA 4, 6, and 22 had reached the chronic stage of bovine besnoitiosis and SA 8 was identified as subclinically infected.

**Figure 2:**
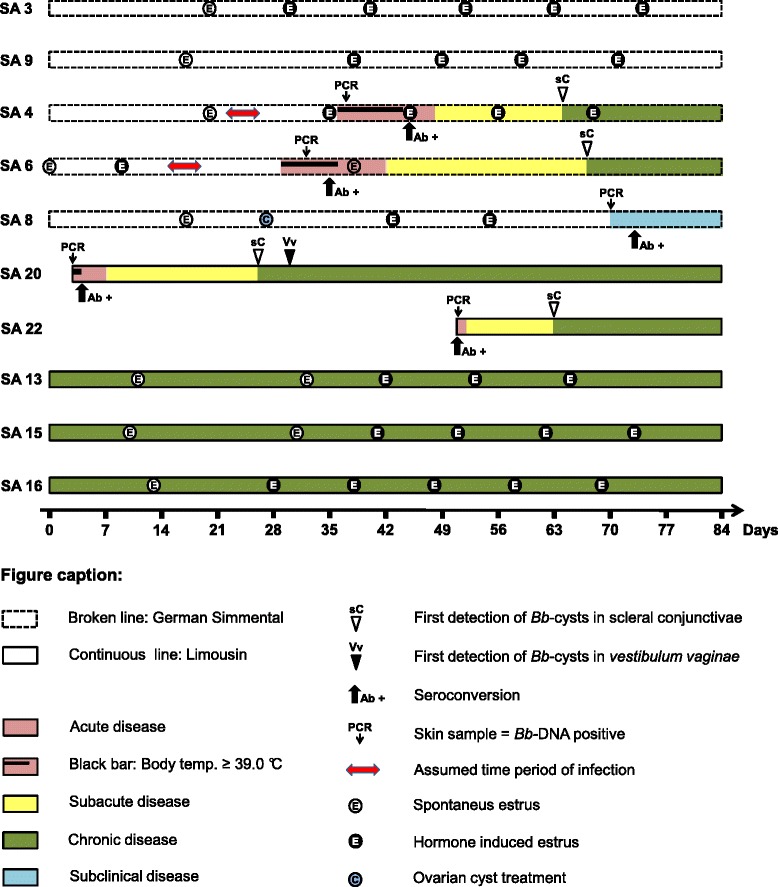
**Chronology of events during the 12-week cohabitation period.** Clinical, serological, and PCR findings in female animals of the pasture group are shown. The acutely *Besnoitia besnoiti* (Bb)-infected Limousin cows SA 20 and 22 were introduced into the experiment on trial days 3 and 51, respectively. Simmental heifers SA 4, 6, and 8 became infected with *B. besnoiti* during the experimental period. In the reference system used, a blood sample was regarded as seropositive if it revealed a positive result in at least two of the three tests applied: IFAT, tachyzoite and bradyzoite immunoblot.

Only Simmental heifers SA 4, 6, and 8 kept in direct contact with clinically affected cattle became *B. besnoiti* infected. SA 1 (bull), 3, and 9, kept under the same conditions and the six control females remained free of *B. besnoiti* infection (Figure 1). Overall, five female cattle were followed for at least 152 days (SA 8) to a maximum of 221 days (SA 20) after first detection of *B. besnoiti* antibodies. SA 4, 6, 20, and 22 became clinically infected. In case of SA 8, only routine laboratory tests revealed an infection with *B. besnoiti*. Skin samples tested *B. besnoiti* DNA positive on td 70 (cycle threshold [ct]-value: 39.1) and SA 8 seroconverted on td 73 (Figure 2) [34]. *B. besnoiti* tachyzoites could not be demonstrated in blood smears of SA 8.

Duration of the acute and subacute stages and the beginning of the chronic stage varied between study animals. To compare diagnostic characteristics between cattle, the time course of infection was adjusted to the day of seroconversion (based on the reference system). For the correlation of clinical findings with laboratory results, periods of time were defined at which SA 4, 6, and 20 were most likely in a particular stage of clinical disease: Acute disease: +/− 3 *dps*; subacute disease: 7 to 18 *dps*; chronic disease: 32 to 152 *dps*. For better comparability of results, these periods of time were also applied to SA 8 and SA 22. In cases where more than two test values were available for the respected clinical periods, the mean of real-time PCR ct-values and the mean of reciprocal IFAT titers were determined for each animal (Table 2).

**Table 2:** **Serology and real-time PCR results based on stage of bovine besnoitiosis** ^**a**^

**Animal data**						
Animal ID		SA 4	SA 6	SA 8	SA 20	SA 22
Breed		S	S	S	L	L
Age (months)		20	19	13	53	49
Pregnancy status		np	np	np	p	p
Study entry (td)		1	1	1	3	51
**Acute disease**						
Start/End (*dps* ^b^)		−9/2	−6/6	subclinical	unknown/4	unknown/0
Ct (+/− 3 *dps*)^c^	Lowest/highest/mean (# sample)	32.5/36.7/33.9 (4)	30.4/36.0/33.8 (7)	39.1/ND/ND (1)	29.5/31.4/30.3 (5)	31.5/ND/ND (1)
IFAT (+/− 3 *dps*)	Lowest/highest/mean (# sample)	100/400/260 (5)	50/400/229 (7)	50/800/ND (2)	800/1600/1280 (5)	400/400/ND (2)
**Subacute disease**						
Start/End (*dps*)		3/18	7/31	subclinical	5/22	1/11
Ct (7–18 *dps*)^c^	Lowest/highest/mean (# sample)	34.5/45.0/ND (2)	33.6/35.3/ND (2)	45.0/45.0/45.0 (3)	28.0/32.9/ND (2)	31.6/ND/ND (1)
IFAT (7–18 *dps*)^d^	Lowest/highest/mean (# sample)	800/1600/1120 (5)	400/1600/867 (6)	800/800/800 (3)	1600/1600/ND (2)	1600/ND/ND (1)
**Chronic disease**						
Start (*dps*)		19	32	subclinical	23	12
Ct (32–152 *dps*)^c^	Lowest/highest/mean (# sample)	45.0 /45.0/45.0 (4)	23.2/37.6/27.6 (5)	27.4/45.0/41.5 (9)	15.5/18.8/16.9 (4)	11.6/19.5/15.3 (3)
IFAT (32–152 *dps*)	Lowest/highest/mean (# sample)	1600/3200/2200 (8)	800/3200/1829 (7)	800/1600/1244 (9)	1600/6400/3556 (9)	6400/6400/6400 (5)

^a^Abbreviations: SA = study animal; S = Simmental; L = Limousin; np = not pregnant; p = pregnant; td = trial day; *dps* = days post seroconversion; ND = not determined; PCR = polymerase chain reaction; IFAT = immunofluorescent antibody test; Ct = Cycle threshold in BbRT2 PCR [30].

^b^Data on the date of seroconversion are previously published [29].

^c^For determination of mean ct-values, negative real-time PCR results were depicted at cycle threshold [Ct] = 45.0.

^d^For SA 22 only laboratory results between 7 to 11 *dps* were regarded.

### Acute stage of bovine besnoitiosis

**Figure 3:**
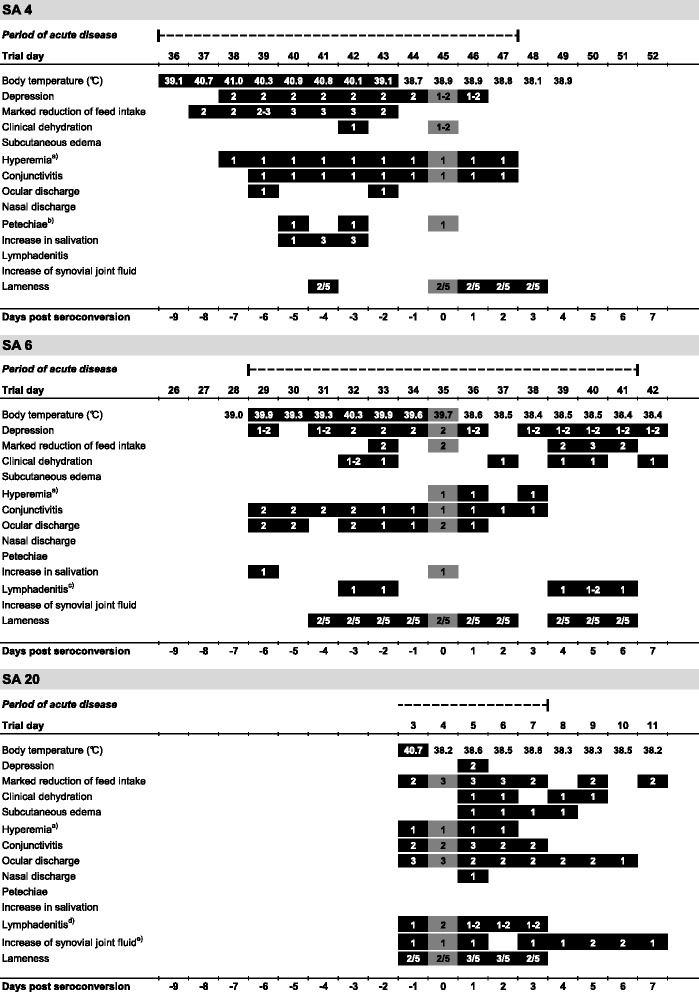
**Clinical findings in acute bovine besnoitiosis in study animals [SA] 4, 6, and 20.** The exact period of acute disease was determined retrospectively after serology results confirmed an infection with *Besnoitia besnoiti*. The acute stage is defined as the period in which an animal had a body temperature of > 39.0°C or showed at least two of the following clinical signs/diagnoses: depression, conjunctivitis, subcutaneous edema, lymphadenitis, lameness. A black bar indicates on which day an animal showed specific clinical alterations during the acute stage of disease. The day of seroconversion is marked by dark grey. The scores 1 (mild), 2 (moderate), and 3 (severe) indicate the severity of alteration from physiological values. For lameness evaluations a five-point scoring system was utilized: Normal gait (1), mild (2), moderate (3), moderate to severe (4), and severe lameness (5) [28]. ^a)^Hyperemia of unpigmented skin and mucous membranes; ^b)^ *Petechiae* in the nasal and oral mucous membranes; ^c)^Lymphadenitis of *Lnn. cervicales superficiales*; ^d)^Lymphadenitis of *Lnn. cervicales superficiales* and *Lnn. subiliaci*; ^e)^Increase of synovial joint fluid in the stifle, carpal and tarsal joints.

**Figure 4:**
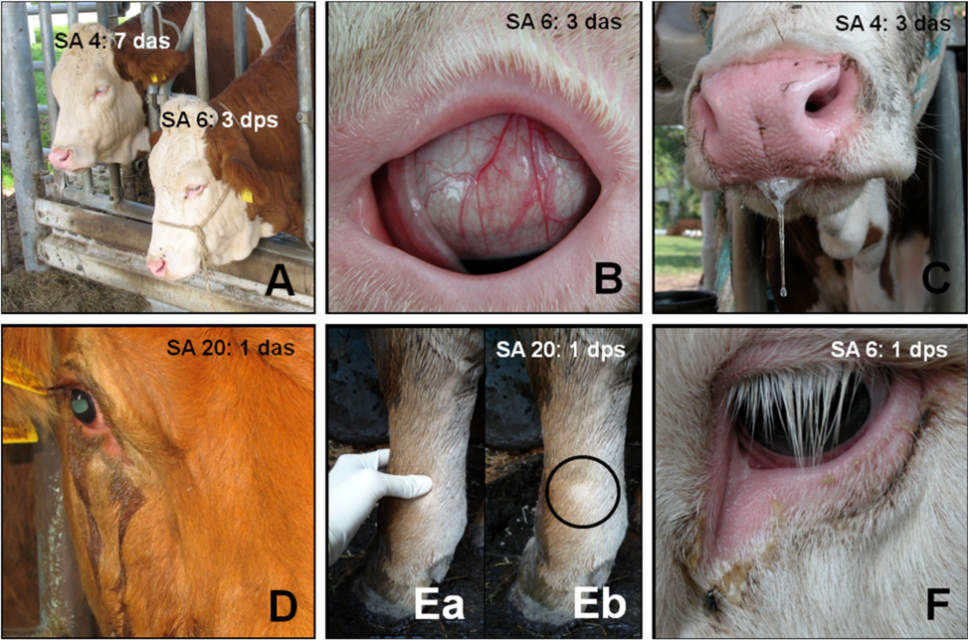
**Study animals [SA] 4, 6, and 20 in acute stage of bovine besnoitiosis.** Animal ID and day when picture was taken are provided in the figure (*das* = days ante seroconversion, *dps* = days post seroconversion). **A)** Depression and inappetence. **B)** Injected scleral vessels and considerable dehydration (sunken eye bulb). **C)** Increase of salivation. **D)** Serous ocular discharge. **E)** Pitting edema on distal left front limb. Note the indentation after applying pressure to the metacarpal skin (Eb: Black circle). **F)** Mild purulent ocular discharge.

The period of acute bovine besnoitiosis of SA 4 and 6 lasted for 12 and 13 days, respectively (Figure 3). *B. besnoiti* DNA could be demonstrated in skin samples of SA 4 and 6, one and four days after the beginning of the acute phase, respectively (SA 4: ct-value: 38.7; SA 6: ct-value: 35.0) (Figure 2) [34]. Limousin cow SA 20, tested positive for *B. besnoiti* DNA in skin samples on the day of admission (ct-value: 30.3) and seroconversion was noted one day later (Figures 2 and 3) [34]. Clinical findings during the acute stage for SA 4, 6 and 20 are shown in Figures 3 and 4.

On the day of admission (td 51), SA 22 had a body temperature of 38.7°C. The Limousin cow was severely depressed, had mucopurulent nasal discharge and showed mild to moderate conjunctival hyperemia and moderate injection of episcleral and conjunctival vessels. The animal walked reluctantly with a very stiff gait on td 51 and 52. Further, *B. besnoiti* DNA could be detected in skin samples (ct-value: 31.5) and the animal had already seroconverted (Figure 2) [34]. According to our definition, SA 22 entered the study on the last day of acute disease. Hereafter, for comparability with other study animals, td 51 will be referred to as day of seroconversion for SA 22.

Disorders of the cardiovascular, respiratory, and digestive systems were not observed in all four clinically affected cattle.

Examinations of blood smears for SA 4, 6, 20, and 22 prepared on days of acute disease did not reveal free or intracellular tachyzoites in peripheral blood. Further serological and real-time PCR results are detailed in Table 2.

### Subacute stage of bovine besnoitiosis

**Figure 5:**
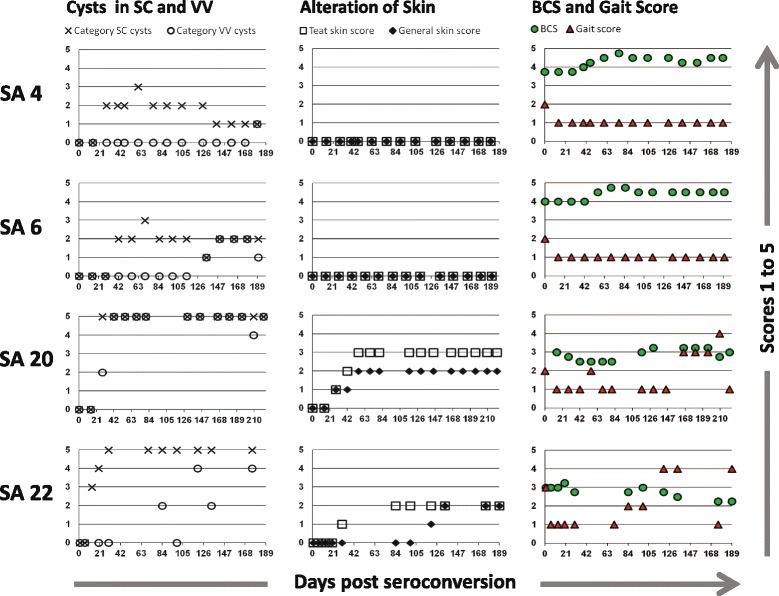
**Selected clinical scores for study animals [SA] 4, 6, 20, and 22.** Changes over time with regards to the number of cysts in the scleral *conjunctivae* and *vestibulum vaginae*, clinical alterations of teat skin and general skin, and changes in body condition and gait are depicted. Scores 1 (mild), 2 (moderate), and 3 (severe) were assigned to characterize the degree of alteration from physiological values of the teat skin (**□**) and general skin (♦). The body condition score (green ●) on a scale of 1–5 in 0.25 increments was determined according to Edmonson et al. [27,31]. For gait alterations (red ▲) a five-point scoring system was utilized following the lameness scoring system by Sprecher et al. [32]: Normal gait (1), mild (2), moderate (3), moderate to severe (4), and severe lameness (5). The number of cysts in the mucous membrane of the *vestibulum vaginae* (**O**) and mean number of cysts in the scleral *conjunctivae* (**x**) were estimated and results were assigned to the following categories: 1: 1–5 cysts; 2: 6–10 cysts; 3: 11–20 cysts; 4: 21–30 cysts; 5: >30 cysts.

Subacute disease, i.e. the phase between the end of acute disease and the macroscopic observation of first tissue cysts, lasted 15, 24, 18, and 10 days for SA 4, 6, 20, and 22, respectively (Figure 2). No abnormal clinical findings were recorded in SA 4 and 6 during the subacute period. In SA 20 and 22, the only alteration noted was a rough and dull hair coat (SA 20: 2, 9, 15, 16 and 19 *dps*; SA 22: 8 *dps*). Good ruminal fill and no decrease in BCS was recorded for SA 4, 6, 20, and 22 (Figure 5).

Serological and real-time PCR results are detailed in Table 2.

### Chronic stage of bovine besnoitiosis

Chronic besnoitiosis led to macroscopical changes of the scleral *conjunctivae* and *vestibula vaginae* in all clinically affected animals. Further, in severely affected cattle SA 20 and 22, effects on body condition, skin and claw health were noted. Serological and real-time PCR results are detailed in Table 2.

#### Clinical changes of mucous membranes and skin

First parasitic cysts in the scleral *conjunctivae* were detected macroscopically 19 and 32 *dps* in SA 4 and 6, respectively. In the *vestibulum vaginae,* parasitic cysts appeared 180 and 135 *dps* for SA 4 and 6, respectively. Throughout the study, the skin of both animals remained clinically unaffected (Figure 5).

**Figure 6:**
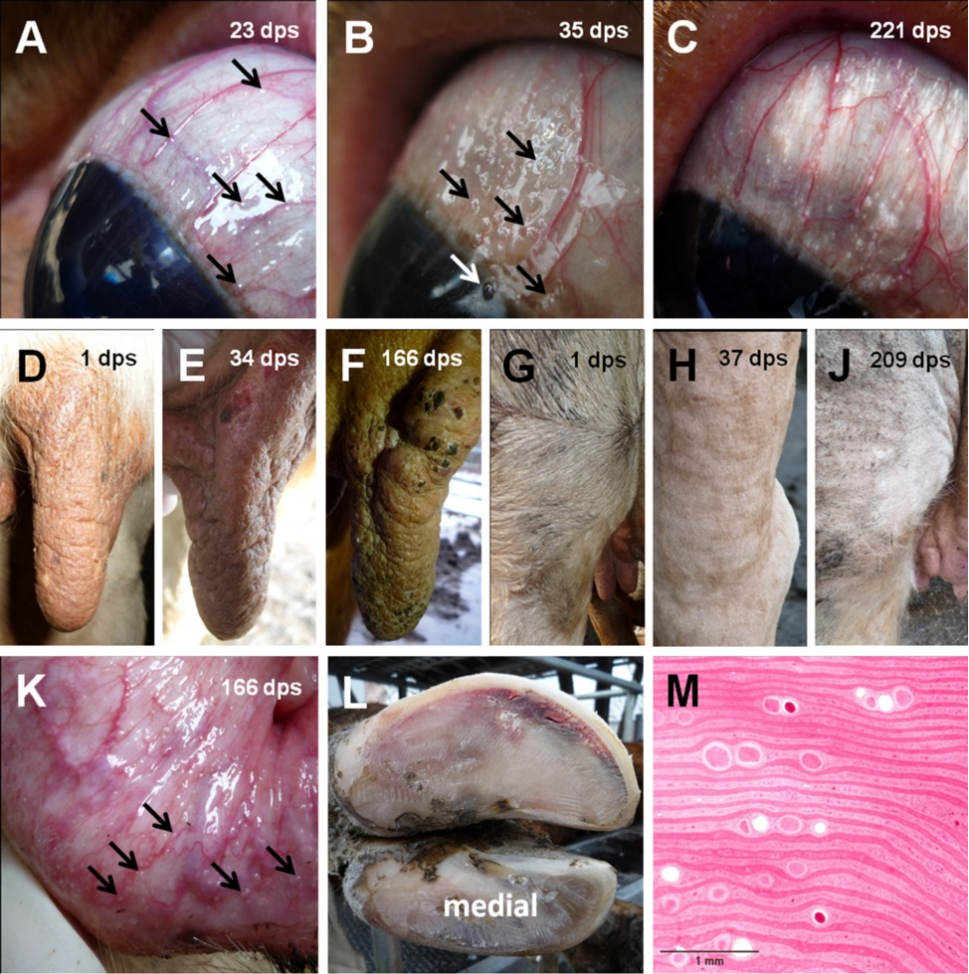
**The chronic stage of bovine besnoitiosis: Clinical findings in study animal 20.** Reference to the day when the picture was taken is provided in the figure (*dps* = days post seroconversion). **A)** Parasitic cysts in the scleral *conjunctivae* were very difficult to detect in the early stage of chronic disease. Only reflections of light where scleral conjunctiva is slightly uneven can be appreciated. A few reflections are indicated (→). **B)** Less than two weeks later, pale, almost translucent cysts were easily noticeable. A few cysts are indicated by the arrow (→). **C)** At the end of the follow-up period cysts were pearl white with an estimated diameter of up to 1 mm. **D)** Clinically unaffected skin of left front teat. **E)** Palpable indurations and open skin lesion on left front teat five weeks later. **F)** Intradermal knots and encrusted epidermal areas on left front teat. **G)** Clinically unaffected skin of left hind limb. **H)** Skin in left tarsal region uneven and slightly lichenified. **J)** Thickend and hypotricheic skin on left hind leg. **K)** Parasitic cysts in the *vestibulum vaginae* were of similar size and color as those in the scleral *conjunctivae* near the end of the follow-up period. A few cysts are indicated (→). **L)** Claws of right hind limb after trimming 176 *dps*. Signs of chronic laminitis indicated by widening of white line and blood stained sole horn of lateral claw. **M)** Multiple mature *Besnoitia besnoiti* tissue cysts in the laminar corium displacing laminar epidermal infoldings (Sample taken at necropsy).

In SA 20 first, very minute parasitic cysts were observed in the scleral *conjunctivae* of both eyes 23 *dps* (Figure 6A) and first parasitic cysts became visible in the mucous membrane of the *vestibulum vaginae* 26 *dps.* The number of visible parasitic cysts in the scleral *conjunctivae* and *vestibulum vaginae* exceeded 30 (Category 5) within 28 *dps* and 42 *dps*, respectively (Figure 5). Palpable indurations of 3–5 mm in diameter in the skin of the teat basis were detected for the first time 23 *dps* (Figure 5). Open skin lesions on the teats were recorded 32 *dps* (Figure 6E). Intradermal knots especially at the teat basis increased in size (up to 1 cm in diameter by 116 *dps*) and open skin lesions as well as encrusted epidermal areas were noted throughout the follow-up period (Figure 6F). The skin of the proximal part of the hind legs was first recorded to be slightly, yet notably uneven 25 *dps* (Figure 6H). These skin alterations became more prominent with time and by 166 *dps* intradermal indurations were palpable and clearly visible on the eyelids, neck, proximal hind legs (Figure 6J), and the distal limbs. The skin of the proximal hind legs was noted to be hypotricheic (Figure 6J) and partial alopecia was found on the distal limbs.

In SA 22, first parasitic cysts were noted in the scleral conjunctivae and *vestibulum vaginae* 13 and 84 days after the end of the acute stage, respectively. First palpable changes were recorded for the teat skin 30 *dps* and for the skin of the upper hind legs and neck 120 *dps* (Figure 5).

#### Effects on body condition

During the chronic stage, ruminal fill was recorded as “good” at all status examinations for SA 4, 6, 20 and 22. Weight gain was recorded for SA 4 and 6, while SA 20 and 22 lost body condition (Figure 5).

#### Claw disease associated with B. besnoiti infections

For SA 4 and 6, lameness was not recorded any time after the acute stage of disease (Figure 5).

SA 20 presented moderate changes in gait and stance 166 *dps*. The animal walked with an arched back, keeping its head low during movement. All limbs were clearly abducted during the protraction phase. At rest, the animal presented a wide stance in both front and hind limbs. The head was kept low and the cow appeared depressed. Functional claw trimming of all feet revealed signs of chronic laminitis on all four lateral claws (Figure 6L) and sole ulcers on both lateral front claws. Keeping the animal on deep straw bedding did not lead to improvement of its overall condition. Shortly after, SA 20 delivered a healthy calf 212 *dps*, a second claw treatment was performed, and the animal was transferred to pasture where lameness receded (Figure 5).

Eighty-four *dps*, SA 22 presented mild supporting leg lameness on the left hind leg. Further, it walked carefully and stiffly on hard concrete surface. A lameness score of 4 was recorded 120 *dps* (Figure 5). The protraction phase of both hind limbs was shortened. While standing, the animal shifted its weight from one hind leg to the other a few times per minute. During movement, front feet were placed carefully, and the head of the animal was kept low. Functional claw trimming of the hind claws revealed chronic laminitis and Rusterholz sole ulcers on the lateral claws. After claw treatment, the animal was housed on deep straw bedding and delivered a healthy calf 152 *dps*. On the last day of the follow-up period, SA 22 was examined on pasture and was found to be free of lameness (Figure 5).

After the follow-up period ended, SA 20 and 22 were rejoined with Herd-BbGer1. However, when signs of severe lameness reappeared, both cattle were submitted to necropsy. Examination of the claws of SA 20 and 22 revealed moderate to severe rotation of the distal phalanges. In several claws, maceration of claw horn was present, and in few claws, a purulent and necrotizing inflammation of the laminar corium and the distal phalanx. Histological examination of the laminar corium revealed multiple *B. besnoiti* tissue cysts leading to distension of the laminar papillae and lamellae and deviation or displacement of the laminar epidermal infoldings (Figure 6M) [33].

### Cattle and insect behavior

Intense mating activity of SA 1 was noted whenever a heifer or cow in the pasture group, irrespective of disease status, was in spontaneous or hormone-induced estrus. With the exception of SA 6 and 9 on td 38, Simmental heifers were not served by the bull on the same day as cows in the chronic stage of bovine besnoitiosis (see Figure 2 for details). Physiological grooming and reproductive behavior of cattle were observed throughout the study. It is noteworthy that animals licked each other’s skin biopsy wounds until potential residual bleeding subsided. Insects were frequently observed feeding on ocular and nasal secretions and on skin wounds after sampling. Healthy cattle reacted to biting insect activity by stamping, kicking, head shaking, frequent tail flicking and contractions of the *musculus cutaneus trunci*. In animals with acute bovine besnoitiosis, reactions to ward off insects were less distinct and numbers of insects on these cattle were noticeably greater than on healthy animals.

### Insect species collected

The following species of the family *Muscidae* (Diptera) were caught on td 24 and 32: Two secretophagous insect species *Musca domestica* Linnaeus 1758 (td 24: n = 40; td 32: n = 2), and *Musca autumnalis* De Geer 1776 (td 24: n = 23; td 32: n = 2); and two hematophagous insect species *Haematobia irritans* Linnaeus 1758 (td 24: n = 10; td 32: n = 34), and *Stomoxys calcitrans* Linnaeus 1758 (td 24: n = 0; td 32: n = 48). One *S. calcitrans*, caught directly on SA 6 during acute besnoitiosis tested positive for *B. besnoiti* DNA in two repetitive examinations (ct-value: 29.99).

## Discussion

In this paper, we present results of the first longitudinal clinical study of cattle monitored in acute, subacute and chronic stages of naturally acquired bovine besnoitiosis in which clinical findings could be correlated with results of current state-of-the-art laboratory tests. The reported 12-week cohabitation experiment of six healthy Simmentals kept with five clinically affected Limousins (three chronic cases, two recently infected animals) resulted in one subclinical case and two mild cases with clinical signs observed in the acute and chronic stage of disease. Major differences between the study described here and a previous cohabitation study [2], are the duration of the experiment (12 weeks compared to 2 years), the frequency of animal sampling, a clinically apparent acute stage, the opportunity to confirm infection after only a few days with serological and molecular techniques, and disease progression monitored in detail.

### Clinical findings

Authors studying the clinical signs of experimentally and naturally acquired acute bovine besnoitiosis report that pyrexia is one of the first signs to appear in the acute stage. Shortly after that anorexia, polypnoe, hyperemia of unpigmented skin and mucous membranes, conjunctivitis, and photophobia develop [1,8,21]. In this study, pyrexia developed one day before (SA 4) or together (SA 6) with the appearance of other clinical signs/diagnoses typical for bovine besnoitiosis, like anorexia, depression, conjunctivitis, and ocular discharge (Figure 3). The duration of pyrexia was comparable with the data obtained by Bigalke (1968) [2], where cattle were ‘naturally’ infected via insects harboring parasites or nasal administration of skin suspensions containing cysts. Interestingly, the hallmark feature of acute bovine besnoitiosis, subcutaneous edema, was only observed in SA 20. As SA 4 and 6 both displayed edema in histological skin sections as well [33], edema in these cases were most likely too subtle to be noticed clinically. *Petechiae*, which are described in experimental bovine besnoitiosis [8] were only observed on a few days in SA 4 (Figure 3). As SA 20 and 22 both displayed multifocal hemorrhages in histological skin sections [33], *petechiae* in these cases were most likely obscured by skin pigmentation. Only SA 20, severely affected by *B. besnoiti*, displayed a palpable increase in synovial joint fluid (Figure 3), which is indicative of arthritis, a lesion which is described in experimental bovine besnoitiosis as well [8].

In the chronic stage of bovine besnoitiosis, all animals developed typical minute parasitic cysts in the non-intestinal mucous membranes. Interestingly, in all four cases, cysts in the scleral *conjunctivae* appeared before cysts in the *vestibula vaginae* became visible (Figure 5). Typical skin lesions associated with bovine besnoitiosis like thickening, folding and reduced elasticity [1,6] developed only in SA 20 and 22.

Cysts in the scleral *conjunctivae* developed 28 and 38 days after first fever in SA 4 and 6, respectively, which is twelve respectively two days shorter compared with the mean reported in Bigalke’s ‘natural’ infections [2]. Interestingly, measurement of cyst diameter in histology revealed smaller cysts around the time point of first detection of cysts in the scleral *conjunctivae* than reported by Bigalke (1968) [2,33]. The course of besnoitiosis in this study reflected those of South African ‘natural’ cases, as SA 4 and 6 developed mild and SA 20 and 22 fairly severe besnoitiosis.

Clinical signs of chronic laminitis during chronic bovine besnoitiosis were only noted in SA 20 and 22. Although lameness or reluctance to move are common clinical signs during acute bovine besnoitiosis [1,21], an association between chronic bovine besnoitiosis and chronic laminitis has only recently been made [33]. Steric interference of tissue cysts with epidermal lamellae and dermal vessels most likely propagates development of chronic laminitis. Inappropriate weight distribution and secondary changes like rotation of the third phalanx and reduction in horn quality then facilitate the formation of lesions like (Rusterholz) sole ulcers. Persistence of parasitic cysts in the dermis followed by chronic inflammation may explain why sole ulcers did not heal in SA 20 and 22 even after appropriate treatment.

Disease stages of bovine besnoitiosis are often not clearly defined in the literature. In accordance with many other authors, we use the onset of pyrexia and other common clinical findings such as depression, conjunctivitis, subcutaneous edema, lymphadenitis, and lameness to define the acute stage [1,12,21]. As scleroderma is a striking feature in chronic besnoitiosis, but not very common in mildly affected animals [2], we used the typical cysts in the scleral *conjunctivae* as determinant of the chronic stage, which is widely accepted and a common tool to detect infected animals and assess disease severity [1,2,22].

### Correlation of clinical findings and laboratory test results

Results of serological and PCR examinations correlated with the course of bovine besnoitiosis in SA 6, 8, 20, and 22 (Table 2). Not surprisingly, IFAT and real-time PCR results reflected the massive parasitism in SA 20 and 22 which is in accordance with previous findings [22,36]. From a 25 mg skin sample 100 μl DNA was extracted. Hereafter, 1 μl was used for PCR. Based on previously published titrations and the assumption that a single parasite contains about 0.01 pg DNA, the number of parasites/25 mg biopsy were estimated [36]. It is possible to estimate that during the acute stage of infection SA 20 and 22 contained the DNA of between ~10^3^ (equals ct-value 32.0) and ~10^4^ (equals ct-value 29.0) parasites/25 mg of biopsy [36]. By contrast, in SA 4, less than the DNA of 10^3^ parasites/25 mg skin sample (equivalent to ct-values > 32.0) was detectable during the acute stage. In SA 8 (ct-value 39.1) even less than 10^1^ parasites/25 mg sample were found. As shown by Langenmayer et al. [33], tachyzoites disappear until the end of the acute stage and the number of cystozoites increases due to intracystic multiplication of *B. besnoiti.* During that stage, parasite DNA per 25 mg sample in SA 20 and 22 remained almost constant (ct-values between 29.5 and 31.5, ~10^3^ - 10^4^ parasites per 25 mg). However, in the remaining animals with low parasite DNA skin levels in the acute stage, there was a clear decrease in the specific DNA levels during the subacute stage. Finally during the chronic stage of disease in SA 20 and 22, the mean estimate increased up to about 10^7^ – 10^10^ parasites per 25 mg sample (ct-values between 11.6 and 19.5).

Although PCR suggests only a mild infection (ct-values between 23.2 and 37.6; ~ 10^1^-10^6^ parasites/25 mg sample) in the chronic stage, SA 6 showed a similar pattern in IFAT as SA 20 and 22. The reciprocal IFAT titer increased over time. Further, in this animal, the estimated number of parasites per 25 μg sample increased from below 10^3^ in the acute stage up to 10^6^ in the chronic stage (minimum ct-value: 23.2). By contrast, SA 4, which showed similar clinical signs as SA 6 during the acute stage, appeared to have successfully eliminated the parasites from the skin over time (no PCR positive skin samples in the chronic stage). This is most probably because of a pronounced immune reaction indicated by high specific antibody titers and early recognition and destruction of dermal cysts [33], leaving only few cysts behind for bradyzoites to proliferate. This pattern is further reflected by the decrease in the number of cysts found in the scleral *conjunctivae* (Figure 5), and supports findings of other studies where clinically infected animals reduce parasite load over time and remain parasite carriers [22]. Interestingly, in subclinically infected SA 8, real-time PCR of skin samples revealed that despite PCR negative results during the subacute stage, and mostly PCR negative results during the chronic stage, up to an estimate of ~10^4^-10^5^ parasites per 25 mg sample (minimum ct-value: 27.4) could be found in 3 of 9 samples. This supports the hypothesis that even subclinically infected cattle may contribute to the propagation of the parasite [22].

It is not clear which factor influenced the high parasite load in two animals (SA 20 and 22) during the chronic stage compared with the remaining animals. Since SA 20 and 22 were the animals with slightly higher parasite loads in the acute stage, it is tempting to speculate that higher loads in the acute stage of infection are determinant for extreme parasite loads in the chronic stage. To confirm this hypothesis and to identify the triggers, further studies analyzing cattle in both the acute and the chronic stage of infection are needed.

A potential effect of meloxicam treatment on disease progression/zoite proliferation is open to debate. However, at present, there are no such studies suggesting such an effect. In this study, Meloxicam treatment of animals was required due to animal welfare regulations (see Ethical statement). Its use most likely affected clinical signs and the sickness behavior response of affected cattle; an effect on parasite propagation, however, is most likely minor.

### Disease transmission

It was beyond the scope of this study to determine routes of parasite transmission and to identify the source of *B. besnoiti* in acutely infected Simmentals. It has previously been demonstrated that *B. besnoiti* can be transmitted mechanically by hematophagous insects and also directly between cattle via the naso-pharyngeal route [2]. In this context a few possible interpretations of the circumstantial data are worth mentioning: Considering the results of PCR and histological examinations [33,34], study animals in the subacute and chronic stage exposed numerous parasitic cysts in biopsy wounds when skin samples were taken at least every other day. Thus, uptake of parasites by naïve cattle licking such wounds and by hematophagous and secretophagous insects feeding on blood and ichor may be presumed. Caught on SA 6 on the day after seroconversion, one out of 48 *S. calcitrans* specimen tested positive for *B. besnoiti* DNA. This further supports the hypothesis that this insect species may contribute to mechanical *B. besnoiti* transmission in the field.

Interestingly, SA 4 and 6 became clinically infected, when only SA 20 (in subacute stage) was frequently biopsied (Figure 2). SA 8, however, became only subclinically infected, even though four cattle in the subacute and chronic stage were frequently sampled during the assumed period of infection (> td 45). The chance for oral uptake of parasites had to be considerably higher for SA 8 compared with SA 4 and 6 (Figure 2). However, insect activity had substantially declined after td 45 when SA 8 became infected (data not shown), which fosters the hypothesis that mechanical transmission by insects may play a role in *B. besnoiti* transmission [2,37,38].

It has been suggested that close contact of cattle is pivotal for insect transmission [2,20,39]. This is supported by the finding that none of six similarly managed and sampled heifers kept at a minimal distance of 20 m to infected cattle seroconverted (Figure 1).

It is widely accepted, despite the lack of well controlled studies, that reproductive activity of cattle may represent a risk factor for parasite transmission. Some authors report that especially bulls become infected with the parasite in *B. besnoiti* affected herds [20,40], and that males may suffer from a more severe course of disease [15,26,41,42]. However, Bigalke (1968) could not confirm an effect of sex with regards to disease incidence [2]. In this particular study, reproductive activity of cattle could not be identified as a factor of parasite propagation. Chronically infected Limousins and SA 4, 6, and 8 had not been simultaneously in estrus during the respective assumed time of infection; the bull SA 1 remained healthy (Figures 1 and 2).

In beef cattle herds, a single mature bull may serve up to 60 cows, often facilitating sexual encounters with multiple females on the same day [43]. This may not only increase the risk of indirect transmission of *B. besnoiti* but also represent an increased hazard for trauma to the mucous membranes of the bull’s penis and prepuce. In this study, only eight females were served by bull SA 1. Even though the reproductive cycles of females were shortened by inducing estrus, there were periods of 24 hours and more when no heifer or cow was in heat (see Figure 2), thus the overall mechanical stress to the bull’s penis and prepuce was low. Additionally, it has to be considered that encounters with chronically affected cows presenting parasitic cysts in the mucous membranes of their *vestibulum vaginae* were only observed on 17 of 84 days (Figure 2).

## Conclusions

This longitudinal study provides detailed results of clinical examinations, serological and PCR tests performed on cattle in all stages of naturally acquired severe, mild and subclinical bovine besnoitiosis. It was demonstrated that recently infected animals can easily be identified by testing blood serum for antibodies twice within a 28 day interval by IFAT and/or western immunoblot. It was further shown that spatial separation of *B. besnoiti* infected and naïve cattle by at least 20 meters may minimize the risk for agent transmission. Reciprocal IFAT titers and parasite DNA loads in skin samples corresponded well with the clinical course of bovine besnoitiosis, representing an important means for herd screening tests and assessment of severity of disease. It was shown that bovine besnoitiosis-associated laminitis represents an important complication in severe chronic disease, which severely impairs animal welfare.

## Additional file

Additional file 1:
**Disease history of chronically** ***Besnoitia besnoiti*** **infected study animals [SA] 13, 15, and 16: Supplementary table providing results of clinical, serological, PCR, and histological examinations of three chronically** ***Besnoitia besnoiti*** **infected, non-pregnant Limousin cows used in the cohabitation trial.**

## Abbreviations

SA: Study animals
td: Trial day
*dps*: Days post seroconversion
Herd-BbGer1: First cattle herd diagnosed with cases of bovine besnoitiosis in Germany
