# Supplementary material for: Natural Besnoitia besnoiti infections in cattle: chronology of disease progression
Source: BMC Vet Res. 2015 Feb 14;11:35. doi: 10.1186/s12917-015-0344-6 (PMC4357170; doi:10.1186/s12917-015-0344-6)
Supplement: Additional file 1: — Disease history of chronically Besnoitia besnoiti infected study animals [SA] 13, 15, and 16: Supplementary table providing results of clinical, serological, PCR, and histological examinations of three chronically Besnoitia besnoiti infected, non-pregnant Limousin cows used in the cohabitation trial. [file 12917_2015_344_MOESM1_ESM.pdf]

Additional file 1: Disease history of chronically *Besnoitia besnoiti* infected study animals [SA] 13, 15, and 16[a]

		SA 13			SA 15			SA 16		
	**Date of exam**	11/08/08	04/01/09	08/17/09	11/08/08	04/01/09	08/17/09	11/08/08	04/01/09	08/17/09
**Clinical signs**	**Mean number of cysts in SC**	n > 50	n > 50	n > 50	pos.[e]	neg.	n = 6-10	n > 20	n > 50	n > 50
	**Number of cysts in VV**	n > 50	n > 50	n > 50	pos.[e]	neg.	n = 11-20	n > 50	n > 50	n > 50
	**Alterations of skin**[b]	++	++	++	+	+	+	++	++	++
**Antibody detection**	**IFAT titer**	1:3200	1:6400	1:6400	1:3200	1:800	1:1600	1:12800	1:6400	1:1600
	**Immunoblot Bradyzoites**[c]	10	10	10	10	10	9	10	10	9
	**Immunoblot Tachyzoites**[c]	10	9	9	10	10	10	10	10	10
**Parasite detection**	**Histology**	pos.	n.i.	pos.	neg.	n.i.	pos.	pos.	n.i.	pos.
	**PCR-Skin**[d]	18; 16	n.i.	n.i.	28	n.i.	n.i.	23; 23	n.i.	n.i.
	**PCR-VV**[d]	24	n.i.	n.i.	24	n.i.	n.i.	27	n.i.	n.i.

[a]Abbreviations: SC = scleral *conjunctivae*; VV = *vestibulum vaginae*; IFAT = immunofluorescent antibody test; PCR = polymerase chain reaction; n.i. = not investigated.

[b] Scoring system: + = Moderate thickening of teat skin with some knot formation, mild thickening of skin around the eye and muzzle, and in the inguinal, perianal and femoral regions; ++ = Severe thickening of teat skin with considerable knot formation, moderate thickening of skin around the eyes and muzzle, on the neck, legs, and in the inguinal, perianal and femoral regions.

[c] Numbers of *Besnoitia besnoiti* tachyzoite and bradyzoite specific bands.

[d] Rounded cycle threshold values; one or two tests conducted.

[e] Quantification of cysts was not conducted.
